# Epidemiology, molecular virology and diagnostics of Schmallenberg virus, an emerging orthobunyavirus in Europe

**DOI:** 10.1186/1297-9716-44-31

**Published:** 2013-05-15

**Authors:** Virginie Doceul, Estelle Lara, Corinne Sailleau, Guillaume Belbis, Jennifer Richardson, Emmanuel Bréard, Cyril Viarouge, Morgane Dominguez, Pascal Hendrikx, Didier Calavas, Alexandra Desprat, Jérôme Languille, Loïc Comtet, Philippe Pourquier, Jean-François Eléouët, Bernard Delmas, Philippe Marianneau, Damien Vitour, Stéphan Zientara

**Affiliations:** 1 ANSES-INRA-ENVA, UMR 1161 Virologie, 23 avenue du Général De Gaulle, Maisons-Alfort 94704, France; 2 Ecole Nationale Vétérinaire d’Alfort, Unité de Pathologie du Bétail, Université Paris-Est, 7 avenue du Général de Gaulle, Maisons-Alfort 94704, France; 3 Direction scientifique des laboratoires, Plateforme de surveillance épidémiologique en santé animale, ANSES, 23 avenue du Général De Gaulle, Maisons-Alfort 94704, France; 4 ANSES-Laboratoire de Lyon, Unité Epidémiologie, 31 avenue Tony Garnier, Lyon cedex 7 69364, France; 5 Direction Générale de l’Alimentation, Ministère de l’Agriculture, 251 Rue de Vaugirard, Paris Cedex 15 75732, France; 6 ID VET, 167 rue Mehdi Ben Barka - Zone Garosud, Montpellier F-34070, France; 7 INRA, Unité de Virologie Immunologie Moléculaires UR892, Jouy-en-Josas F-78350, France; 8 ANSES-Laboratoire de Lyon, Unité Virologie, 31 avenue Tony Garnier, Lyon cedex 7 69394, France

## Abstract

After the unexpected emergence of Bluetongue virus serotype 8 (BTV-8) in northern Europe in 2006, another arbovirus, Schmallenberg virus (SBV), emerged in Europe in 2011 causing a new economically important disease in ruminants. The virus, belonging to the O*rthobunyavirus* genus in the *Bunyaviridae* family, was first detected in Germany, in The Netherlands and in Belgium in 2011 and soon after in the United Kingdom, France, Italy, Luxembourg, Spain, Denmark and Switzerland. This review describes the current knowledge on the emergence, epidemiology, clinical signs, molecular virology and diagnosis of SBV infection.

## Table of contents

1. Introduction

2. Timeline of SBV infection in Europe

3. Molecular virology

3.1. Genome and structure

3.1. Life cycle

3.1. Pathogenicity

3.1. Phylogeny

4. Clinical signs

5. Transmission

6. Origin

7. Diagnosis

8. Surveillance

9. Seroprevalence

10. Impact

11. Prevention

12. Conclusions

13. Competing interests

14. Author’s contributions

15. Acknowledgements

16. References

## 1. Introduction

At the end of the summer and in the autumn 2011, hyperthermia and drop in milk production were reported in adult dairy cows in north-west Germany and The Netherlands [1]. In some cases, transient diarrhoea was also recorded in the Netherlands [2]. Some of the symptoms observed were similar to the disease caused by Bluetongue virus (BTV) and a re-emergence of this virus that led to a major epizooty in 2006–2008 in Europe was feared. Surprisingly, no known bovine pathogen was identified in samples from symptomatic cattle [3-5]. In November 2011, the Friedrich-Loëffler Institute (FLI) in Germany detected viral RNA belonging to a new virus in a pool of blood samples from clinically affected dairy cows using a metagenomic approach [3]. This new virus was called Schmallenberg virus (SBV) after the place of origin of the collected samples. Analysis of viral genomic sequences revealed similarities with Akabane, Aino and Shamonda viruses, all belonging to the *Orthobunyavirus* genus from the *Bunyaviridae* family. Douglas, Sathuperi and Shamonda viruses were later identified as closer relatives of SBV [6]. A specific real-time quantitative reverse transcription PCR (RT-qPCR) was then developed by FLI to detect the SBV genome and the protocol shared with many European partners. The inoculation of 9-month old calves with blood of cattle that were RT-qPCR positive for SBV or with the virus isolated in *Culicoides variipennis* larvae cells (KC cells) caused fever and mucous diarrhoea, providing experimental evidence that SBV might be responsible for the clinical signs observed [3].

This paper reviews current knowledge on the emergence, molecular virology, clinical signs, diagnosis and seroprevalence of SBV and is based on data published up to the end of January 2013 in peer-reviewed journals, internet-based reporting systems such as the Program for Monitoring Emerging Diseases (proMED-mail), communications from research institutes and official reports from governmental and European institutions such as the European Food and Safety Authority (EFSA).

## 2. Timeline of SBV infection in Europe

SBV was first detected in Germany and The Netherlands in 2011 [3]. In December 2011, The Netherlands reported a teratogenic effect of SBV in sheep with the birth of malformed lambs with crooked neck, hyrocephalus and stiff joints [2]. The presence of SBV was then reported in Belgium at the end of December 2011 and in the United Kingdom on the 22^nd^ of January 2012. France reported its first case of SBV on the 25^th^ of January 2012 after the virus genome was detected by RT-qPCR in brain samples from malformed lambs born on farms located in the territorial divisions of “Moselle” and “Meurthe et Moselle” in north-eastern France [7]. The presence of SBV was then reported in Luxembourg on the 16^th^ of February [8]. On the 17^th^ of February, SBV was confirmed in a malformed goat in north-east Italy [8] and on the 12^th^ of March, in Spain (Andalusia), in a newborn lamb [9].

By the end of April 2012, SBV had been detected in 3628 herds in Europe [10]. SBV-infected holdings recorded up to this date corresponded to infections occurring in 2011. In May 2012, acute SBV infections were detected in cattle in south west France in the Pyrénées-Atlantiques territorial division [11], indicating that SBV was able to re-circulate after the winter period. Similar conclusions were also made after the detection of the virus in the United Kingdom in newborn lambs born in May and June 2012 [12,13] and in Germany in cattle, sheep and goat holdings sampled in 2012 [14].

Early 2012, the development of assays to detect anti-SBV antibodies, as discussed later in this review, provided a useful tool to show evidence of SBV infection since viraemia is transient [3,15].

On the 5^th^ of June, Denmark reported the presence of antibodies against SBV in two cattle from southern Jutland [16] and on the 20^th^ of July, Switzerland confirmed its first cases of acute SBV infection in cows from two farms in the canton of Berne [17].

By August 2012, more than 5500 cases of SBV infection in ruminants had been recorded across northern Europe [18].

In mid-September, anti-SBV antibodies were detected in Austria in cattle and sheep [19]. At the beginning of October 2012, the presence of antibodies to SBV was reported in western Poland in goats that were sampled at the end of July 2012 [20,21] and in Sweden in cows [22]. In mid-October, anti-SBV antibodies were detected in northern Scotland in a tup and in two cows from Finland that were sampled at the end of September 2012 [23]. Further studies suggested that the virus had spread to South Finland during the summer and early autumn of 2012 [24]. At the end of October 2012, the presence of SBV was detected in Ireland in a bovine foetus [25,26] and a few days later, in Northern Ireland in a malformed calf [27,28].

By the end of October 2012, SBV infection was confirmed by RT-qPCR and/or serology in approximately 6000 holdings in Europe [29].

In November 2012, antibodies against the virus were detected in milk from cattle herds in Norway [30] and an outbreak of SBV was reported in Italy (Sardinia) in a sheep flock with cases of abortion and foetal malformations [31]. At the end of December 2012, SBV was detected for the first time in the Czech Republic following the birth of malformed lambs [32]. In mid-January 2013, the first cases of SBV were confirmed in Estonia in sheep foetuses [33] and at the end of January the presence of SBV was confirmed in sheep in Slovenia [34].

All these reports show that SBV has spread rapidly in vast parts of Europe.

## 3. Molecular virology

Analysis of viral sequences has led to the classification of SBV in the *Bunyaviridae* family and the *Orthobunyavirus* genus. The *Bunyaviridae* family is composed of 350 viruses that are divided into 5 genera: *Orthobunyavirus, Hantavirus*, *Nairovirus*, *Phlebovirus* and *Tospovirus*. Viruses from this family infect vertebrates with the exception of tospoviruses that are plant viruses. Viruses such as Rift Valley fever virus (*Phlebovirus*), Akabane virus (*Orthobunyavirus*) and Nairobi sheep disease virus (*Nairovirus*) are important pathogens in veterinary medicine. Other viruses, such as hantaviruses responsible for hemorrhagic fever with renal syndrome and cardio-pulmonary syndrome or Crimean*-*Congo hemorrhagic fever virus (*Nairovirus*), can cause serious disease in humans. The *Orthobunyavirus* genus is composed of more than 170 viruses divided into 18 serogroups. SBV belongs to the Simbu serogroup that also includes Simbu virus, Oropouche virus, Akabane virus, Douglas virus, Sathuperi virus, Aino virus, Shamonda virus, Peaton virus and many others.

### 3.1. Genome and structure

**Figure 1:**
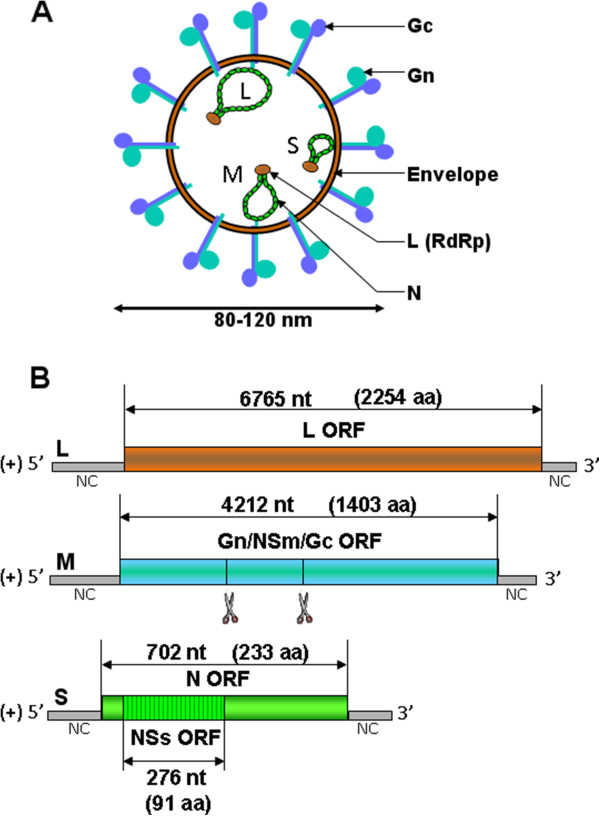
**Schematic representation of a generic bunyavirus virus particle (A) and SBV antigenomes (B). (A)** The bunyavirus virion has a diameter of 80 to 120 nm. The three RNA segments (S, M and L) associate with the L polymerase and the N nucleoprotein to form RNP. **(B)** The three antigenomic RNA encode for several predicated ORF as indicated by double-sided arrows. The number of nucleotides (nt) of the different ORF and the corresponding number of amino acids (aa) are shown [3]. Putative co-translational cleavage sites of the polyprotein encoded by segment M are indicated by scissors but are not yet characterised.

The bunyavirus genome consists of 3 segments of negative-sense single-stranded RNA: the L (large), M (medium) and S (small) segments [35] (Figure 1A). Phleboviruses and tospoviruses differ from other bunyaviruses since their S segment adopts an ambisense coding strategy. The L segment encodes the RNA-dependent RNA polymerase (RdRp) L (or L protein), the M segment encodes a precursor polyprotein that is co-translationally cleaved into the envelope glycoproteins Gn and Gc and the non-structural protein NSm and the S segment encodes the nucleoprotein N and the non-structural protein NSs in an overlapping open reading frame. The three segments of the SBV genome have been fully sequenced [3,36] but its structure and the different encoded proteins are not yet well-characterised and can only be predicated from the data available on related viruses (Figure 1B).

Virions from bunyaviruses are enveloped, spherical and have a diameter of approximately 80 to 120 nm. They acquire their membrane when budding at the Golgi apparatus lumen [37-39]. Electron microscopy performed at the FLI confirmed that, similarly to other bunyaviruses, the SBV virus particle has a diameter of approximately 100 nm and is membrane-enveloped [40]. Virus particles of bunyaviruses are constituted of 4 structural proteins: the two surface glycoproteins Gn and Gc and the viral polymerase complex composed of the polymerase L protein and the nucleoprotein N. This complex is responsible for the transcription and replication of the viruses that occur exclusively in the cytoplasm. Inside the virus particle, the viral genome is present as a ribonucleoprotein (RNP) associated with many copies of the nucleoprotein N and a few copies of the polymerase L.

### 3.2. Life cycle

Since SBV was discovered recently, very little is known on its life cycle and knowledge accumulated on other bunyaviruses can only be discussed.

The replication cycle of bunyaviruses is exclusively cytoplasmic. It begins with the recognition of the cellular receptor, unknown for the majority of bunyaviruses, by the Gn/Gc heterodimers present at the surface of the virion membrane [41,42]. Virions enter the cell via endocytosis. Change of pH in vesicles induces conformational modifications of the viral glycoproteins and the exposure of the Gc fusion peptide [43]. The viral envelope fuses with the membranes of the endosomes and the RNP are released inside the cytoplasm. The primary transcription can start and produce viral mRNA via a mechanism of catch-snatching [44]. The L protein cleaves a sequence of 10 to 18 nucleotides from the 5’ end of capped mature cellular mRNA to use it as a primer for the initiation of viral transcription. Viral mRNA are synthesised and translation by host cell ribosomes leads to the production of viral proteins. The L and N proteins are needed for replication of the viral genome and the glycoproteins Gn and Gc form heterodimer complexes in the endoplasmic reticulum. Both glycoproteins are then transported to the Golgi apparatus via a Golgi-retention signal, located on Gn for most bunyaviruses, where their glycosylation is completed [45].

Assembly of bunyaviruses is thought to occur mostly in tubular virus factories containing both cellular and viral components situated around the Golgi complex [37,46,47]. From free nucleotides, the viral L polymerase produces complementary copies of the whole viral genome called antigenomes that are also present as RNP and are needed for the production of high quantities of viral genomes. Newly-formed genome RNP then accumulate in the Golgi complex where they directly interact with the C-terminal domains of the glycoproteins Gn and Gc [48-51]. Maturation of viral particles occurs via budding through the modified membrane of the Golgi apparatus. For orthobunyaviruses, these steps depend on the NSm protein that is associated with the Golgi apparatus via three transmembrane domains [52,53]. Mature virus particles are then transported in vesicles to the plasma membrane where they are released in the extracellular compartment by exocytosis. Further morphological changes then occur resulting in the release of fully infectious extracellular virus particles.

### 3.3. Pathogenicity

The pathogenicity of orthobunyaviruses is dependent on multiple viral factors encoded by the three genomic segments. For example, the neuroinvasive ability of La Crosse virus, another orthobunyavirus from the California serogroup, is determined by the polymerase and/or the glycoproteins whereas the host immune response is inhibited by NSs that antagonises the expression of type I interferon (IFN-I) and the transcription mediated by RNA polymerase II [54-59]. NSs from Bunyamwera (Bunyamwera serogroup), the prototype virus for the *Orthobunyavirus* genus and the *Bunyaviridae* family, also contributes to viral pathogenesis and seems to be a major virulence factor. This non-structural protein inhibits protein synthesis and the host cell antiviral response by interfering with RNA polymerase II-dependent transcription, IFN-I production and apoptosis mediated by IRF-3 [60-65].

Although there is no conservation of sequence, NSs from other bunyaviruses are also involved in pathogenesis and inhibition of the host cell antiviral response. For example, NSs from Rift Valley fever virus suppresses host transcription by interfering with the subunits of the transcription factor II H (TFIIH) complex, degrades dsRNA-activated protein kinase (PKR) and represses the activation of the promoter IFN-β via its association with a subunit of the Sin3A repressor complex [66-70].

However, little is known about the viral factors involved in the pathogenicity of viruses involved in veterinary medicine, such as Akabane virus, Shamonda virus and SBV. Recently, it was shown that IFN-I receptor (IFNAR) knock-out mice [71] are susceptible to SBV infection and can develop fatal disease as previously reported for La Crosse virus [72] and that intracerebral injection of SBV is lethal for NIH-Swiss mice [36]. Moreover, a study has suggested that infectious serum from cattle is more suitable for standardised SBV infection model than culture-grown virus [73]. These models could be useful in the future to study SBV pathogenesis and contribute to the design of vaccines. Reverse genetic systems have also been developed for SBV and provide a powerful tool to characterise the virus [36,74]. Recombinant viruses lacking NSs have already been generated to study the role of the viral protein as a virulence factor. It was subsequently shown that NSs is not essential for the virus to replicate in vitro [36,74] but a virus lacking the viral protein is attenuated in newborn mice [36]. NSs was found to block protein synthesis [74] and to interfere with the production of IFN [36,74] suggesting that, similarly to other bunyaviruses, NSs of SBV is able to modulate the host innate immune response.

### 3.4. Phylogeny

Initial phylogenetic analysis of the SBV genomic segments indicated that SBV displays 69% identity with Akabane virus for the L segment, 71% identity with Aino virus for the M segment and 97% identity with Shamonda virus for the S segment [3]. After analysis of additional sequence data, it was reported that the M segment of the Sathuperi and Douglas orthobunyaviruses display higher identity with SBV whereas the S and L segments are closer to the Shamonda virus [75]. All these viruses belong to the Simbu serogroup, although no cross-protection has been reported between them. All together, these studies suggested that the 3 genomic segments of SBV could be the result of a reassortment between the segment M of the Sathuperi virus and segments S and L of the Shamonda virus. Nevertheless, another group has recently determined the almost full-genome sequences of 9 viruses from the Simbu serogroups belonging to five species (i.e. species *Shamonda virus* (Shamonda virus, Peaton virus and Sango virus), species *Sathuperi virus* (Douglas virus and Sathuperi virus), species *Shuni virus* (Aino virus and Shuni virus), species *Akabane virus* (Sabo virus) and species *Simbu virus* (Simbu virus)) [6]. Phylogenetic analysis of all of these sequences has shown that SBV belongs to the *Sathuperi virus* species. This conclusion is also supported by a serological investigation showing that Douglas and Sathuperi viruses, but not Shamonda virus, are neutralised by anti-SBV serum [6]. In this study, it was also suggested that SBV is an ancestor of Shamonda virus, the latter being a reassortant containing the S and L genomic segments from SBV and the M segment from an unclassified virus.

## 4. Clinical signs

Sheep and goats seem to be very mildly affected by SBV infection. Symptoms are more apparent in adult cows, and include loss of appetite, hyperthermia and diarrhoea, which can lead to a 50% reduction in milk production [76-78]. Symptoms usually disappear within a few days. The viraemia induced by SBV is short-lived, lasting for 2 to 6 days in cattle [3,78].

**Figure 2:**
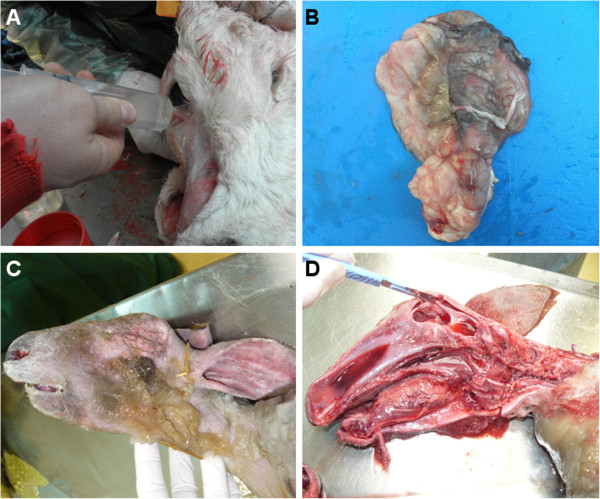
**Clinical manifestations of SBV.** Necropsy of a three-day-old SBV-positive calf suffering from amaurosis and hydranencephaly **(A-B)** and a SBV-positive stillborn lamb presenting arthrogryposis and hydranencephaly **(C-D)**. **(A)** The cerebral hemispheres were replaced by a fluid-filled sac containing 250 mL of cerebrospinal fluid (hydrocephaly) that was removed with a syringe. **(B)** The cerebral hemispheres were examined after removal of the cerebrospinal fluid. The cerebellum was present but appeared hypoplastic. **(C)** Brachygnathia was observed in the stillborn lamb. **(D)** The remaining cerebral hemispheres appeared as a 3 mm thick wall enclosing cerebrospinal fluid. The brainstem was preserved but not the cerebellum.

In December 2011, The Netherlands reported a teratogenic effect of SBV in sheep with manifestations comparable to those observed for Akabane and Aino viruses [79-82]. Infected females are able to transmit the virus to foetuses (ovine, caprine and bovine), which developed atypical malformations leading most frequently to intra-uterine death or death immediately after birth. Common congenital malformations and clinical signs in aborted and stillborn animals include a neuro-musculo-skeletal disorder called arthrogryposis, severe torticollis, ankylosis, kyphosis, lordosis scoliosis, brachygnathia inferior and neurological disorders such as amaurosis, ataxia and/or behavioral abnormalities (“dummy syndrome” as observed during the epizooty caused by BTV serotype 8 (BTV-8) [83-85] (Figure 2).

In case of twin gestation, one twin can suffer from arthrogryposis and the other from neurological disorders. One twin can also be born malformed and the other one viable or only affected by a delayed growth.

Newborns suffer from severe neurological disorders that generally lead to death of the animal several hours to several days after birth. It was reported that a SBV-positive one-week old calf born at term showed severe central nervous system lesions, severe dysfunctions of the cerebral cortex, basal ganglia and mesencephalon, severe porencephaly or hydranencephaly but no arthrogryposis [86]. Interestingly, the SBV genome was still detectable in the CNS, suggesting that it is able to persist in the infected foetus after birth.

Necropsy has revealed some cases of hydranencephaly (lack of the brain cerebral hemispheres), hydrocephaly, cerebral and cerebellar hypoplasia and porencephaly [84,86-88] (Figure 2).

Histological studies have revealed lymphohistiocytic inflammation in the central nervous system and glial nodules in the mesencephalon and hippocampus in ovine species. Astrogliosis and microgliosis were detected in both calves and lambs and some cases of myofibrillar hypoplasia of skeletal muscles were reported for both species [84]. Histological examination of the brain and spinal cord of a ten-day old SBV RT-qPCR-positive calf has also reported the presence of meningoencephalitis and poliomyelitis [89]. Furthermore, immunohistochemistry and in situ hybridisation methods performed on brain sections have suggested that neurons are the major target for SBV replication in naturally infected newborn lambs and calves [36,88].

In the case of Akabane virus, infection of the foetus occurs between the 28^th^ and 36^th^ day of gestation in sheep, the 30^th^ and 50^th^ day in goats and the 76^th^ and 174^th^ day in bovines [81]. The severity of foetal injury depends on the time of infection during gestation and maximal damage occurs when neuronal tissues are differentiating [78,81,90]. Detection of the SBV genome in brain, blood or spleen samples in malformed aborted or stillborn ruminants, which represent the majority of the diagnosed cases from December 2011 to March 2012 for lambs and March to June 2012 for calves, indicates that infection of the mother occurred during gestation, in autumn 2011 [29]. A study has estimated the risk of SBV infection for foetuses born from cows primo-infected after formation of the placenta to 28% [91]. No visible clinical signs are present at birth if in utero infection occurs while the immune system of the foetus is able to control the infection [91].

SBV can infect bison as reported in Germany [92]. The virus is also able to infect wild cervids and llamas but no clinical signs or macroscopic abnormalities were recorded for these species [93,94]. The virus might infect other wild species and domestic animals such as horses or dogs, as reported for viruses belonging to the *Orthobunyavirus* genus. However, SBV infection has not yet been reported in these species. Most of the viruses from the Simbu serogroup are not considered to be zoonotic, with the exception of Oropouche virus, which can infect humans and provoke severe flu-like symptoms. To date, no evidence of SBV infection in humans has been reported and no SBV-neutralising antibodies have been detected in sera from persons (farmers and veterinarians) exposed to the virus [95,96].

## 5. Transmission

The majority of bunyaviruses are transmitted by arthropod vectors and, in particular, mosquitoes, phlebotoms, culicoides, ticks and thrips, with the exception of hantaviruses which are transmitted by rodents. Studies have shown that viruses within the Simbu serogroup are mostly transmitted by culicoides, but also by mosquitoes from the *Aedes* and *Culex* genus and by several species of ticks [97,98]. Recently, a study has reported the presence of the SBV genome in a pool of culicoides (*C. obsoletus complex*, *C. chiopterus* and *C. dewulfi*) trapped from July to October 2011 in Belgium [99]. Culicoides from the *C. obsoletus* group trapped in Denmark during the same period also contained SBV RNA [100]. Furthermore, SBV RNA was detected in *C. obsoletus complex* and *C. chiopterus* collected in August-September 2011 in the Netherlands where the prevalence of SBV among Culicoides at this period was estimated to be around 0.25% [101]. The virus has also been found in biting midges in Norway, Poland and Sweden [102-104]. These studies suggest that species of culicoides identified as vectors for BTV also act as vectors for the transmission of SBV [105-108]. To date, no studies have been carried out to assess the ability of other arthropods, such as mosquitoes and ticks, to act as vectors for the transmission of SBV.

Cases of acute SBV infection were recorded following the start of the 2012 vector season in France, the United Kingdom, Switzerland, Germany and Italy [11-14,17,29,31]. It is not yet known how SBV is able to persist despite the winter season. It could be the result of the vector population surviving the cold season or the virus persisting in the cattle population or in other reservoirs. It has been reported that some *culicoides* species are present inside farm buildings during the winter and are able to complete their life cycle in animal enclosures [109,110]. It is then possible that SBV is able to persist from year to year in the vector population despite winter temperatures.

## 6. Origin

The history and geographical origins of SBV raise numerous questions. As for the introduction of BTV-8 in The Netherlands and Belgium in 2006, the causes of SBV emergence in northern Europe remain unknown. The closest relatives to SBV are Sathuperi, Douglas, Shamonda, Akabane, Aino, Peaton or Sango viruses but, to date, they have not been identified in Europe [111]. These viruses are present in other parts of the world and seem to be able to emerge in regions far from their areas of enzootic distribution. Akabane and Aino viruses are distributed in the Far East and Australia, and Akabane virus has been found recently in Israel and also in Turkey. Similarly, Shamonda virus was detected in Nigeria in the 1960s and has been isolated since only in Japan in 2005 [112]. Although SBV was discovered only recently, there is no doubt that its origins are more ancient and that it might have co-evolved with other closely related viruses. Viruses belonging to the Simbu serogroup have not been well studied and epidemiological data are poor. Nevertheless, phylogenetic analyses based on samples taken in different regions of the world at different periods of time suggest that these viruses evolve slowly. For example, the nucleotide sequences of Japanese strains of the Shamonda virus isolated in 2005 differ by only 3% from those of strains isolated in Nigeria 30 years before [112].

As discussed above, Shamonda virus is thought to be a reassortant containing the S and L genomic segments from SBV and the M segment from an unclassified virus [6]. Such reassortment phenomena have been described previously between viruses of the *Orthobunyavirus* genus [113]. In the Simbu serogroup, it is thought that Peaton virus is derived from an ancestor generated by a reassortment between Akabane and Aino viruses [114] and that a virus circulating in Japan is the product of reassortment between a Japanese strain of the Aino virus and an Australian strain of the Peaton virus [113].

SBV emergence is a reminder that bunyaviruses and particularly orthobunyaviruses are a potential threat for European livestock. Akabane and Aino viruses are already present in the Mediterranean region and might be introduced into Europe. Consequently, the surveillance of livestock and vector populations is critical to monitor the emergence of such viruses in Europe. The identification of the Batai virus, an *Orthobunyavirus* belonging to the Bunyamwera serogroup, in populations of *Anopheles maculipennis* trapped in the south-western part of Germany in 2009 illustrates the importance of this type of surveillance [115].

It is surprising that SBV has emerged in the same region of Europe as BTV-8 in 2006 and later BTV-6 and −11, and may suggest that the viruses followed the same route of introduction. SBV might have been present before in a region of the world where no and/or rare clinical signs were manifested or reported in the native population. Considering the increase in international trade of animals and products of animal or vegetal origin, it is of no doubt that Europe will face more and more frequently this type of emergence.

## 7. Diagnosis

Diagnosis of SBV infection relies on the detection of the viral genome by RT-qPCR, as mentioned above. It is a duplex assay that was initially developed by the FLI [3,116]. The technique is based on the simultaneous amplification of a SBV gene and an endogenous gene, β-actin or GAPDH, which is used as an internal positive control (IPC) to ascertain RNA integrity and the absence of PCR inhibitors. Primers amplifying a part of the L gene segment were first used as a template for the detection of the SBV genome. A protocol targeting the S segment was then optimised and showed higher sensitivity [15]. This protocol was implemented in most laboratories in Europe. Brain samples from aborted or stillborn lambs, kids and calves have mainly been used for diagnosis of SBV. Studies have shown that samples from the cerebrum, external placental fluid and the umbilical and spinal cord are suitable for the detection of SBV [116] and that the highest concentration of SBV RNA is found in the brainstem [117,118].

Viral isolation requires the inoculation of Vero (African green monkey kidney epithelial), BHK-21 (baby hamster kidney fibroblast) or KC (*Culicoides variipennis* larvae) cells with brain, serum or blood samples.

Once SBV strains were available, virus neutralisation tests (VNT) and a plaque reduction neutralisation test were developed to detect antibodies present in the serum of infected animals [96,119,120]. These methods are time-consuming (4 to 6 days) and cannot be automated. A diagnostic test that allows serological testing of a large number of samples, not possible by VNT, was needed to diagnose SBV infection and estimate SBV seroprevalence in infected areas. An indirect ELISA test based on a recombinant SBV nucleoprotein antigen produced by ID-VET (Montpellier, F-34070) was designed. This test was validated by the Animal Health laboratory at the French agency for food, environmental and occupational health and safety (ANSES) in Maisons-Alfort (Alfort ANSES laboratory) in April 2012 and currently provides a rapid and less expensive tool for serological diagnosis [121]. A Dutch group has developed an ELISA based on SBV that has been cultured on partially purified and completely inactivated Vero cells [122]. This assay was shown to be a sensitive test to detect antibodies in foetal or procolostral sera and diagnose SBV in newborn calves and lambs. ELISA tests have also been developed to detect anti-SBV antibodies in milk [123,124].

Interestingly, in a study looking at anti-SBV antibodies in foetal blood samples from calves and lambs, it was shown that 74% of the samples tested positive for SBV infection by ELISA whereas only 27% were SBV-positive by RT-qPCR [122]. Using a VNT assay [121], the Alfort ANSES laboratory also found that from 71 aborted or stillborn animals with malformations evocative of SBV, for which the laboratories had received brain samples and serum samples from their “mothers”, 100% of the mother sera were VNT positive whereas only 61.9% (44/71) of the offspring brain samples were SBV-positive by RT-qPCR (unpublished data). These different results suggest that the diagnosis by RT-qPCR might have underestimated the real number of SBV cases. It is possible that the foetus is able to produce neutralising antibodies that will clear the virus. Neutralising antibodies against Akabane virus can be found in foetal and precolostral sera from calves [79,125]. This would explain why, in some cases, aborted or stillborn animals with clinical signs of SBV infection are found RT-qPCR-negative for the virus and the difficulties to isolate the virus. Other explanations would be that the genome of the virus is destroyed after the death of the foetus or during sampling and processing of the samples or that SBV infection is not the cause of the malformations observed.

## 8. Surveillance

**Figure 3:**
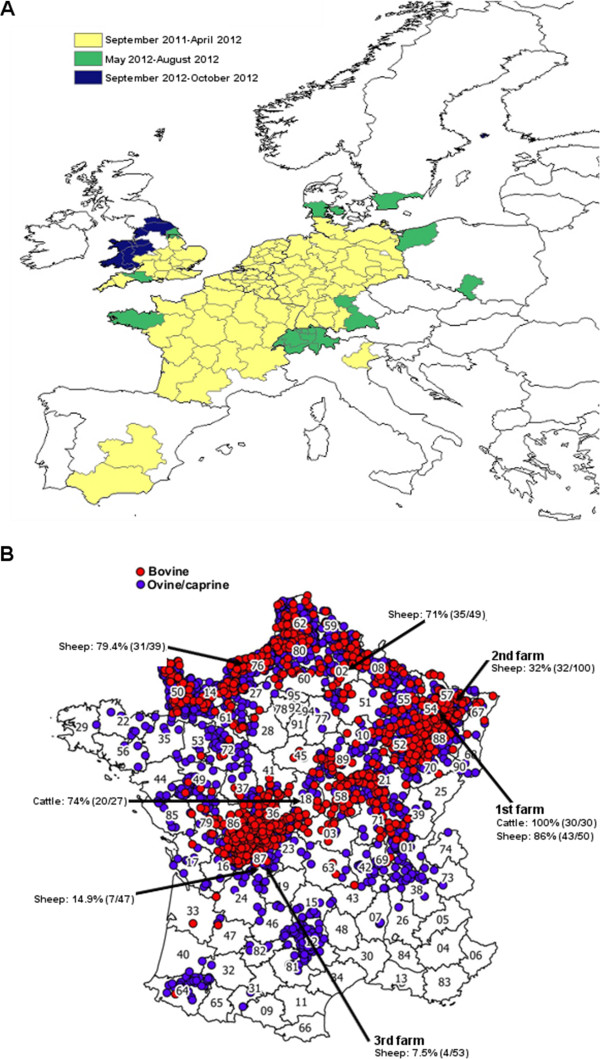
**Location of SBV infection in Europe and France. (A)** Spread of SBV infection for all ruminants in Europe from September 2011 to October 2012 (source: European Food and Safety Authority [29]). Affected countries reported the location and the time of the first report of confirmed SBV infection. **(B)** Location of SBV infected farms in France as of August 31, 2012 (source: French Ministry of Agriculture [127]). A total of 3197 farms have been reported to be SBV-infected. The seroprevalence of SBV in several farms from different French territorial divisions (northern regions (02 and 76), eastern region (54 and 57) and central region (18 and 87)) where SBV had been previously detected or neighbouring farms is also shown [132]. The presence of anti-SBV antibodies in cattle or sheep were detected by ELISA and VNT. The first, second and third farms where SBV was detected in France are also indicated.

Sharing knowledge and tools has allowed affected and neighbouring regions to efficiently and rapidly monitor the progression of SBV infections in Europe as shown in Figure 3A.

For example, in France, a national passive SBV surveillance system coordinated by the Ministry of Agriculture was launched on the 4^th^ of January 2012 in consultation with the French epidemiological surveillance platform for animal health. Veterinarians were asked to send samples (brain, spleen) from stillborns or malformed ruminant offspring showing clinical signs such as arthrogryposis, torticolis, scoliosis, brachygnathia and/or hydranencephaly. RT-qPCR was performed to detect SBV RNA and virus was isolated using the protocols kindly provided by Dr Martin Beer and Dr Bernd Hoffmann from the FLI, as described above [3,116]. From December 2011 to the beginning of March 2012, the Alfort ANSES laboratory was the laboratory designated by the Ministry of Agriculture to diagnose SBV infection in France. In mid-March 2012, a network of 66 local laboratories was established for SBV testing. This laboratory network is similar to the one established for the detection of the BTV genome by RT-qPCR and has considerably increased the capacity to test samples from suspected animals. This structure allowed the testing of hundreds of thousands of blood samples by RT-qPCR or by ELISA using automats. Thus, the experience gained during the BTV-8 emergence in 2006 [126] has facilitated the rapid development of a laboratory network for SBV diagnosis at a national level. The surveillance measures established by the French government ended on the 31^st^ of May for ovine and caprine ruminants and on the 31^st^ of August for bovines. As of the 31^th^ of August 2012, 3197 SBV infected farms had been reported in France (i.e. in which at least one malformed offspring tested positive for SBV by RT-qPCR), including 1143 sheep farms, 2019 cattle farms and 35 goat farms (Figure 3B). SBV-infected farms are localised mainly in the north-east and the central-west of France [127]. Following the new cases of SBV recorded in different regions of France since the beginning of September 2012, surveillance measures were re-established from the 1^st^ of November 2012 to monitor congenital forms of SBV. They will take into account all cases detected from the 1^st^ of September 2012 [128]. To date, France remains the country with the highest number of SBV-infected farms recorded.

## 9. Seroprevalence

Serologic investigations are needed to determine seroprevalence in affected and neighbouring regions and monitor the spread of SBV infection. They are also useful to improve modelling predictions and assess the overall impact of SBV. Serological studies were conducted in several European countries following the design of new tools to detect anti-SBV antibodies.

A study investigating the prevalence of antibodies against SBV among dairy cattle in The Netherlands during winter 2011–2012 has reported an estimated seroprevalence of 72.5% [129].

Antibodies against SBV have been detected in 91% of cows sampled within 250 km of the location where SBV emerged in Belgium in February-April 2012 [91]. Additional studies performed in all of Belgium have reported a between-herd and within-herd seroprevalence of 99.76% and 86.3%, respectively in cattle (11 635 cattle from 422 herds sampled between the 2^nd^ of January and the 7^th^ of March 2012) [130]. A between-herd seroprevalence of 98.03% and a within-herd seroprevalence of 84.31% were also found in sheep (1082 sheep from 83 flocks sampled between the 4^th^ of November 2011 and the 4^th^ of April 2012) and a within-herd seroprevalence of 40.68% was found in goats (142 goats from 8 flocks sampled during the same period) [131]. These reports suggest that most host animals have been in contact with SBV in Belgium.

Preliminary seroprevalence studies were also undertaken by the Alfort ANSES laboratory in farms from different French territorial divisions using ELISA and VNT [132]. A high seroprevalence of SBV in cattle and sheep herds (32 to 100%) was found in northern and eastern regions of France as shown on Figure 3B. SBV seroprevalence seems lower in farms situated in southern parts of France (7.5 to 14.9%). These data are in accordance with the introduction of SBV in France from the north-east part of the country where the first cases were diagnosed. This suggests that, like BTV in 2007, SBV spread from the north-east of France to the rest of the territory. However, more serological studies need to be undertaken to confirm these findings.

The high seroprevalence of SBV found in The Netherlands, Belgium and in several French farms, indicates a widespread exposure to SBV during the biting insect season in 2011. Retrospective and prospective serological studies on ovine, bovine and caprine livestock will help to elucidate the period of SBV introduction in Europe. Similarly, it would be interesting to study the seroprevalence of SBV in wild species to evaluate their involvement as a reservoir of the virus. A study has reported the presence of anti-SBV antibodies in roe and red deer from southern Belgium sampled from October to December 2011 [93]. An average seroprevalence of 43.1% was found in the cervids sampled suggesting that SBV had spread rapidly among wild deer and that wild species could be involved as reservoirs of the virus.

## 10. Impact

SBV has a low or limited impact on animal health. Reports submitted by different European countries affected by SBV have estimated morbidity and mortality rates of less than 3% [133]. The highest proportion of SBV-confirmed herds in comparison with the total number of herds per region is 6.6% for sheep and 4% for cattle [29]. Nevertheless, it remains difficult to provide an accurate estimation of the number of herds affected by the virus and to determine the economic impact of the disease on the livestock industry since cases were most likely underreported or underdetected [132,134,135].

Few data are available on the impact of SBV within herds. A preliminary survey investigating the impact of SBV infection in sheep flocks in France has been conducted recently. This study showed that in SBV RT-qPCR-positive flocks, an average of 85% lambs were healthy while 13% were born dead or died rapidly after birth and 2% were born with malformations but survived for more than 12 h (data from 363 flocks and 64 548 lambs) [136]. However, the clinical impact was very variable between flocks. This might be due to differences in the proportion of ewes at a susceptible stage of pregnancy at the time of exposure to SBV.

## 11. Prevention

Considering SBV has a limited impact on animal health, trade restrictions have not been advised and are regarded as unjustified by the European Union (EU) and the World Organisation for Animal Health (OIE) [137]. SBV is transmitted by a vector that is widespread within Europe and movement bans would be ineffective. However, many countries outside the EU have imposed restrictions on the import of live animals and products from the EU such as semen and embryos. Recently, SBV RNA has been detected in the semen of naturally infected bulls and SBV infection was reported in calves inoculated experimentally with SBV RT-qPCR-positive semen [138-140]. These findings show that the semen of bulls naturally infected with SBV can be infectious and suggest that SBV and Akabane virus differ in terms of semen contamination since a group has reported previously that the semen from bulls infected experimentally with Akabane virus is not infectious [141]. The export of semen from countries where SBV is present might represent a risk of contamination. It is then essential to develop a sensitive test for the detection of SBV RNA in semen and to perform further studies to determine the risk of transmission of SBV via this route and the impact of the virus on fertility.

Vaccination is a preventive measure that could reduce the impact of SBV [142]. The development of vaccines is in progress and a vaccine should be available commercially in the future [30,143]. Nevertheless, the cost of vaccination for the livestock industry might not be justified since SBV seems to be a low impact disease. Vaccines have been developed against Akabane disease but management of outbreaks relies mainly on the sentinel monitoring of vectors and cattle [142,144,145].

## 12. Conclusions

The emergence of SBV at the end of 2011 in Europe is a reminder that the introduction of new diseases remains a threat for European countries. The rapid response to the emergence of SBV established by affected European countries has shown that an efficient network of laboratories is in place to face the emergence of new animal viruses. SBV was able to re-circulate after the winter season and is still circulating in Europe. A certain level of protection exists within the ruminant populations but it is likely that a significant percentage of animals remains susceptible to SBV in areas where no or few cases of SBV have been reported. This suggests that new congenital cases of SBV infection will occur during the winter 2012–2013. Maintaining an efficient surveillance would be essential to further describe the progression of the epidemic and its impact on the breeding industry. More studies are needed to determine the regions in which SBV is present, to understand its geographical and genetic origin and to identify its putative reservoirs. A better understanding of the pathogenesis associated with SBV infection and the ability of SBV antibodies to protect animals against the disease will also be useful to control the disease.

## Competing interests

The authors declare that they have no competing interests.

## Authors’ contributions

VD structured the review and prepared, along with DV, EL and SZ, the draft of the manuscript. CS, GB, EB, JR, CV, MD, PH, DC, AD, JL, LC, PP, JE, BE and PM helped with the French epidemiological, serological and clinical data presented in this paper. All authors read and approved the final manuscript.
